# m^6^A Methyltransferase METTL14-Mediated Upregulation of Cytidine Deaminase Promoting Gemcitabine Resistance in Pancreatic Cancer

**DOI:** 10.3389/fonc.2021.696371

**Published:** 2021-08-11

**Authors:** Congjun Zhang, Shuangyan Ou, Yuan Zhou, Pei Liu, Peiying Zhang, Ziqian Li, Ruocai Xu, Yuqiang Li

**Affiliations:** ^1^Department of Oncology, The First Affiliated Hospital of Anhui Medical University, Hefei, China; ^2^Department of Digestion and Urology, Hunan Tumor Hospital, Changsha, China; ^3^Department of General Surgery, Xiangya Hospital, Central South University, Changsha, China; ^4^Tumor Center, Hunan Chest Hospital, Changsha, China

**Keywords:** N6-methyladenosine, pancreatic cancer, chemotherapy, p65, METTL14

## Abstract

**Objective:**

Pancreatic cancer is one of the most lethal human malignancies. Gemcitabine is widely used to treat pancreatic cancer, and the resistance to chemotherapy is the major difficulty in treating the disease. *N* ^6^-methyladenosine (m^6^A) modification, which regulates RNA splicing, stability, translocation, and translation, plays critical roles in cancer physiological and pathological processes. METTL14, an m6A Lmethyltransferase, was found deregulated in multiple cancer types. However, its role in gemcitabine resistance in pancreatic cancer remains elusive.

**Methods:**

The mRNA and protein level of m^6^A modification associated genes were assessed by QRT-PCR and western blotting. Then, gemcitabine‐resistant pancreatic cancer cells were established. The growth of pancreatic cancer cells were analyzed using CCK8 assay and colony formation assay. METTL14 was depleted by using shRNA. The binding of p65 on METTL14 promoter was assessed by chromatin immunoprecipitation (ChIP) assay. Protein level of deoxycytidine kinase (DCK) and cytidine deaminase (CDA) was evaluated by western blotting. *In vivo* experiments were conducted to further confirm the critical role of METTL14 in gemcitabine resistance.

**Results:**

We found that gemcitabine treatment significantly increased the expression of m^6^A methyltransferase METTL14, and METTL14 was up-regulated in gemcitabine-resistance human pancreatic cancer cells. Suppression of METTL14 obviously increased the sensitivity of gemcitabine in resistant cells. Moreover, we identified that transcriptional factor p65 targeted the promoter region of METTL14 and up-regulated its expression, which then increased the expression of cytidine deaminase (CDA), an enzyme inactivates gemcitabine. Furthermore, *in vivo* experiment showed that depletion of METTL14 rescue the response of resistance cell to gemcitabine in a xenograft model.

**Conclusion:**

Our study suggested that METTL14 is a potential target for chemotherapy resistance in pancreatic cancer.

## Introduction

Pancreatic cancer (PC) is one of the most aggressive and lethal cancer diseases worldwide, with only about 5% of patients survived 5 years after diagnosis (1, 2). The poor outcomes might result from metastatic spread, high incidence of recurrence and chemotherapy resistance (3). The treatments of PC include surgery, chemotherapy, and radiation therapy. Since surgery alone is insufficient to achieve long-term survival, adjuvant chemotherapy is always involved in the treatment regimen of PC patients (4, 5). The current standard chemotherapy treatment regimen includes gemcitabine monotherapy and its combinations with other drugs, such as 5-fluorouracil, capecitabine, and carboplatin et al. (6, 7). Gemcitabine (Gem, 2’,2’‐difluorodeoxycytidine), an anti-metabolite drug, is the first‐line agent used for pancreatic cancer treatment (8). After uptake by cells, gemcitabine is phosphorylated into gemcitabine monophosphate by deoxycytidine kinase (DCK), and then transformed to active forms, gemcitabine diphosphate and triphosphate (9). On the contrary, gemcitabine is inactivated by cytidine deaminase (CDA), which could convert gemcitabine to 2’,2’-difluorodeoxyuridine (10). Although patients with gemcitabine treatment showed some clinical benefit, less than 15% of patients achieved progression-free at 6 months from diagnosis (3, 11). Most patients acquired resistance to gemcitabine, and the mechanism remains largely unknown (8). Thus, investigating the underlying mechanism of gemcitabine resistance in pancreatic cancer is urgently required.

N6-methyladenosine (m^6^A) modifications, one of the most common type of RNA modification, can be found in messenger RNA (mRNA), long non-coding RNA (lncRNA), and microRNA (miRNA) (12, 13). m^6^A regulates mRNA splicing, translation efficiency, and stability, thus regulates the expression of target genes (14, 15). m^6^A modification is a dynamic process that regulated by three categories of genes, which name Writers, Erasers and Readers. The Writers, including methyltransferase-like enzyme 3 (METTL3), methyltransferase-like enzyme (METTL14), RNA binding motif protein 15/15B (RBM15/15B), Wilms tumor 1-associated protein (WTAP), and vir-like m^6^A methyltransferase-associated (VIRMA), are responsible for the methylation process, and the core component is METTL3-METTL14 complex. The Erasers, including fat and obesity-related protein (FTO) and alkB homolog 5 (ALKBH5), act as demethylase enzymes and can reverse methylation. The Readers, which include HNRNPC, YTHDC1, YTHDC2, YTHDF1 and YTHDF2, recognize the m^6^A methylation and generate functional signals (16–19). Accumulating evidence suggested that m^6^A modification closely related to tumor genesis and development (20–23).

Previous studies reported that METTL3, METTL14, FTO, ALKBH5 and YTHDF2 play important roles in pancreatic cancer cells (24–28). In addition, m^6^A methylation was shown to play a crucial role in drug resistance development and intervention in cancer cells (29, 30). Although Taketo et al. reported that METTL3 depletion enhanced chemo, radio, and chemoradio-sensitivity in pancreatic cancer cell (31), the role of m^6^A methylation in gemcitabine resistance remains largely unknown. To address this question, we establish the gemcitabine resistant pancreatic cancer cell lines, and evaluated the expression level of m^6^A modification associated proteins, then systematically assessed their functions in gemcitabine resistance.

## Materials and Methods

### Clinical Samples

Gemcitabine sensitive and resistant pancreatic tumors were obtained from 10 patients (for protein extraction), all resected from August 2016 to August 2020. All tumor were verified as adenocarcinomas. The use of clinical samples was approved by the Human Research Ethics Committee of the First Affiliated Hospital of Anhui Medical University.

### Antibodies and Plasmid

Antibodies against METTL3, METTL14, WTAP, FTO, ALKBH5, p65, p-p65, and GAPDH were obtained from Cell Signaling; antibodies against VIRMA, DCK and CDA were purchased from Proteintech.

### Cell Culture and Reagents

The human pancreatic cancer cell lines PANC-1, BxPC-3, MIA Paca2, Hs 766T, AsPC-1 and Capan-2 cell line were obtained from the American Type Culture Collection (ATCC, USA). PANC-1, MIA Paca2, Hs 766T, and Capan-2 cell lines were cultured in Dulbecco’s modified Eagle’s medium (Gibco, USA) with 10% fetal bovine serum (FBS, Gibco, USA) at 37°C in a humidified 5% CO2 atmosphere incubator. BxPC-3 and AsPC-1 cell lines was cultured in RPMI 1640 medium (Gibco, USA) with 10% fetal bovine serum (FBS). To generate gemcitabine‐resistant pancreatic cancer cells, PANC‐1 and BxPC-3 cells were cultured in medium supplemented with gradually increasing concentrations of gemcitabine (Sigma-Aldrich, USA) for more than 1 month until the cells could expand in the presence of 2000 nM gemcitabine, and named PANC-1 GemR and BxPC-3 GemR.

### Transient Transfection of Small Interfering RNA

Small interfering RNA was purchased from Sigma-Aldrich to deplete p65 proteins. siControl nontargeting siRNA was purchased from Dharmacon. PANC-1 GemR and BxPC-3 GemR cells were transfected with siControl and specific siRNA in 6-well plates at a final concentration of 100 nM using DharmaFECT1 transfection reagent (Dharmacon, USA) according to the manufacturer’s instructions and harvested 72 h later.

### Short Hairpin RNA Knockdown

METTL14 shRNAs were purchased from Sigma-Aldrich and pLKO.1-puro was used as the control plasmid. Recombinant lentiviruses were packaged by co-transfecting shRNA plasmids with packaging constructs according to the manufacturer’s instruction. PANC-1 GemR cells were incubated with lentivirus mixed with 8 μg/ml polybrene for 24 h, replaced fresh medium and incubated for another 48 h. Stable expression of shControl and shMETTL14 were established by puromycin (1 mg/mL) selection.

### Plasmid Overexpression Experiment

p65 overexpression plasmid was purchased from addgene (32). Briefly, pancreatic cancer cells plated onto 6 well plate were transiently transfected with 2 μg empty vector or p65 plasmid using Lipofectamine 2000 transfection reagent (Invitrogen, USA) following the manufacturer’s instructions for 24-48 h.

### Cell Viability

Cell viability was detected by CCK8 assays (Jiangsu KeyGENBioTECH Corp., Ltd, China). Briefly, 3000-5000 cells per well were seeded into 96‐well plates the day before treatment. After incubated with a range of different concentration of gemcitabine for 72 h, 10 μl CCK8 reagent was added into each well and incubated at 37°C for 2 h and then recorded the absorbance at 450 nm with a 96-well plate reader.

### Colony Formation Assays

1000 cells per well were plated in 6-well plates the day before treatment. After treatment, cells were incubated at 37°C for 10-14 days. Then, the cells were fixed, stained with 0.2% crystal violet, and imaged to determine the number of colonies. Clones that consisted of at least 50 cells were counted as one colony.

### RNA Isolation and Quantitative Reverse‐Transcription PCR

Total RNA was isolated using RNeasy Mini Kit (Qiagen, USA) and cDNA was synthesized using script cDNA synthesis kit (Bio-Rad, USA) according to manufacturer’s instructions. Quantitative PCR was performed using SYBR Premix Ex TaqII (TaKaRa, Japan) on CFX96 real-time PCR detection system (Bio-Rad, USA) with the following primers: METTL3, forward: 5’-AGCCTTCTGAACCAACAGTCC-3′, reverse: 5′-CCGACCTCGAGAGCGAAAT-3′; METTL14, forward: 5’-TTTCTCTGGTGTGGTTCTGG-3′, reverse: 5′-AAGTCTTAGTCTTCCCAGGATTG-3′; WTAP, forward: 5’-CAACCT CTTTAGCCAAACAAGAA-3′, reverse: 5′-ATTCCTGAGTGC AACAGC-3; VIRMA, forward: 5’-AAGTGCCCCTGTTTTCGATAG-3′, reverse: 5′-ACCAGACCATCAGTATTCACCT-3′; FTO, forward: 5’-ACTTGGCTCCCTTATCTGACC-3′, reverse: 5′-TGTGCAGTGTGAGAAAGGCTT-3′; ALKBH5, forward: 5’-CGGCGAAGGCTACACTTACG-3′, reverse: 5′-CCACCAGCTTTTGGATCACCA-3′; GAPDH, forward: 5’- AATCCCATCACCATCTTCCAG-3′, reverse: 5′-AAATGAGCCCCAGCCTTC-3′. Values for each gene were normalized to the expression of GAPDH.

### mRNA Stability Analysis

mRNA stability analysis was performed as previously described (33). Briefly, PANC-1 cells infected with scramble or METTL14 shRNA for 72 h were directly harvested or treated with 5 mM Actinomycin D and harvested at the indicated time points. Equal RNA amounts (1 μg) were transcribed into cDNA using the script cDNA synthesis kit (Bio-Rad, USA). Gene expression was analyzed using the SYBRGreen reagent (TAKARA, Japan).

### Western Blot Analysis

RIPA buffer (Invitrogen, USA) supplemented with protease and phosphatase inhibitor cocktail (Sigma-Aldrich) was used to lysed the cell. Protein concentration was determined using a BCA protein assay kit (Pierce, USA). 20 μg total proteins from each sample were subjected to SDS-PAGE separation, and then transferred to a nitrocellulose membrane. After blocked with 5% milk for 1 hour, the membranes were incubated with the primary antibodies at 4°C overnight. Then, the blots were washed three times with TBS-T and incubated with secondary antibodies for 1h at room temperature. Chemiluminescence was detected by exposing the membrane to high performance Super XR film (Fujifilm, PYSER-SGI limited) or using Mini Chemiluminescent Imaging and Analysis System (Sagecreation). The signal was detected using Super Signal West Pico Chemiluminescent Substrate (Thermo Scientific, USA). The densitometric quantification was performed by using Image J software. Data are reported as means ± SEM of values from three experiments.

### Chromatin Immunoprecipitation Assay

ChIP assays were performed using the SimpleChIP^®^ Enzymatic Chromatin IP Kit (Cell Signaling Technologies, USA) as per manufacturer’s instructions. Briefly, 1X107 cells were fixed using 1% formaldehyde for 10 min at room temperature, and the crosslinking process was stopped by adding glycine. After washed twice in ice cold PBS, cells were subjected to nuclei preparation and chromatin digestion. The chromatin was digested using micrococcal nuclease to length of approximately 150-900 bp. The cross-linked chromatin preparation was then diluted and incubated with 2 ug antibody against p65 at 4°C with rotation overnight. Then, 30 µl of Protein G Magnetic Beads was added to each IP reaction and incubate for 2 h at 4°C with rotation, and washed with low salt wash and high salt wash. The chromatin was eluted from the antibody/protein G magnetic beads by placing the tube at 65°C for 30 min, and Proteinase K was added to the eluted chromatin supernatant to reverse cross-links. After purification, the immunoprecipitated DNA and input were analyzed by QRT-PCR using the following primers: p65 binding site 1, forward: 5’- GGACATAAACCAAGACACGCTTT-3′, reverse: 5′- CGCAGGTAACAGCCACAACA-3′; p65 binding site 2, forward: 5’-AAGTACTTACCGCGATTTCCAA-3′, reverse: 5′-TTTCAAGCCTACAGTGGCGG-3′.

### *In-Vivo* Xenograft Experiment

NOD/SCID mice (6-week-old) were injected (subcutaneously in both flanks) with 5.0 x 106 PANC-1 GemR cells (infected with scr or METTL14 shRNA) per mouse suspended in 50 ul PBS and mixed with equal volume of growth factor reduced matrigel. One week after injection, we started measuring tumor size at the indicated times. Tumor size was calculated by 0.5 × (long diameter) × (short diameter)2. The mice were treated with vehicle or 100 mg/kg gemcitabine intraperitoneally twice a week. Tumor weight was measured at the end of the experiment. All animal experiments were conducted according to the NIH Guide for the Care and Use of Laboratory Animals.

### Statistical Analysis

Data were presented as mean± SD from three independent experiments. *P* value was determined using paired Student’s t-test, and a *P value < 0.05 was considered statistically significant.

## Results

### Gemcitabine Treatment Increased the Expression of METTL14

**Figure 1:**
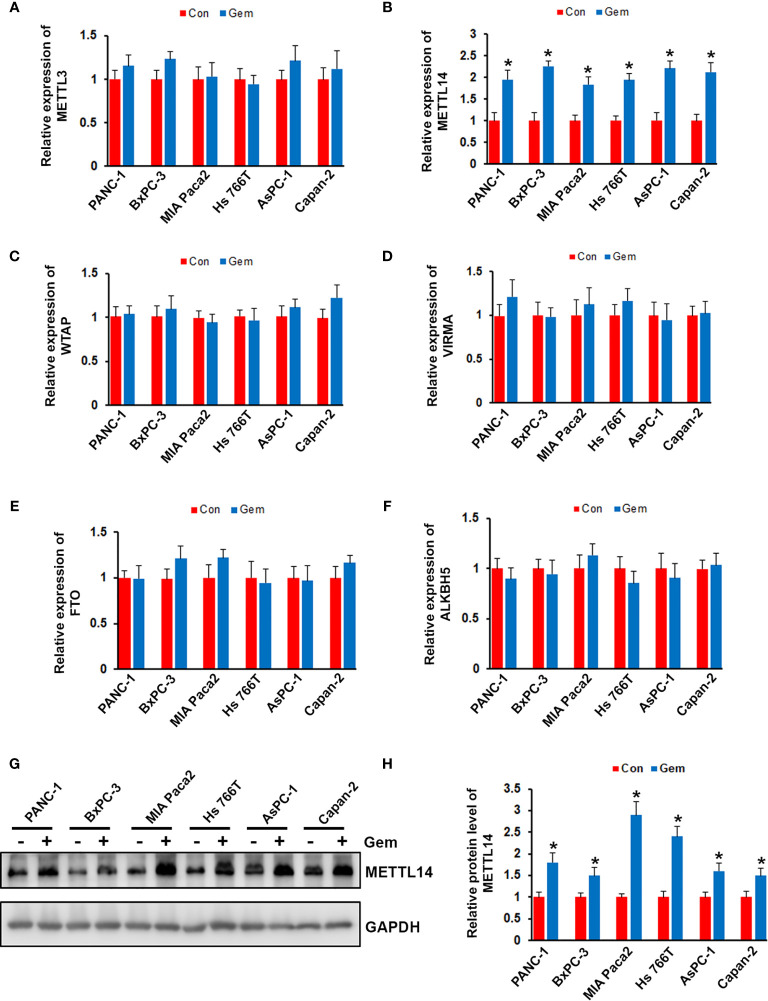
Effect of gemcitabine on METTL14 expression. **(A–F)** Pancreatic cancer cells (PANC-1, BxPC-3, MIA Paca2, Hs 766T, AsPC-1 and Capan-2) were treated with 100nM gemcitabine for 48 hours, the mRNA level of METTL3, METTL14, WTAP, VIRMA, FTO, and ALKBH5 was assessed by QRT-PCR. *p < 0.05. **(G)** Pancreatic cancer cells were treated with 100nM gemcitabine for 48 hours, the protein level of METTL14 was assessed by western blotting. **(H)** Quantification of METTL14 protein levels in pancreatic cancer cells treated with or without gemcitabine. Data are reported as means ± SEM of values from three experiments. *p < 0.05.

To investigate the effect of gemcitabine on the expression of m6A modification associated proteins, we treated the pancreatic cancer cells (PANC-1, BxPC-3, MIA Paca2, Hs 766T, AsPC-1 and Capan-2) with gemcitabine. After treated with gemcitabine, only METTL14 was significantly increased in all six pancreatic cancer cell lines, but not METTL3, WTAP, VIRMA, FTO or ALKBH5 (**Figures 1A–F**). Consistent with the QRT-PCR results, our western blotting analysis also showed that the protein level of METTL14 was increased after gemcitabine treatment (**Figures 1G, H**). These results suggested a potential role of METTL14 in gemcitabine resistance.

### METTL14 Was Overexpressed in Gemcitabine Resistant Pancreatic Cancer Cells

**Figure 2:**
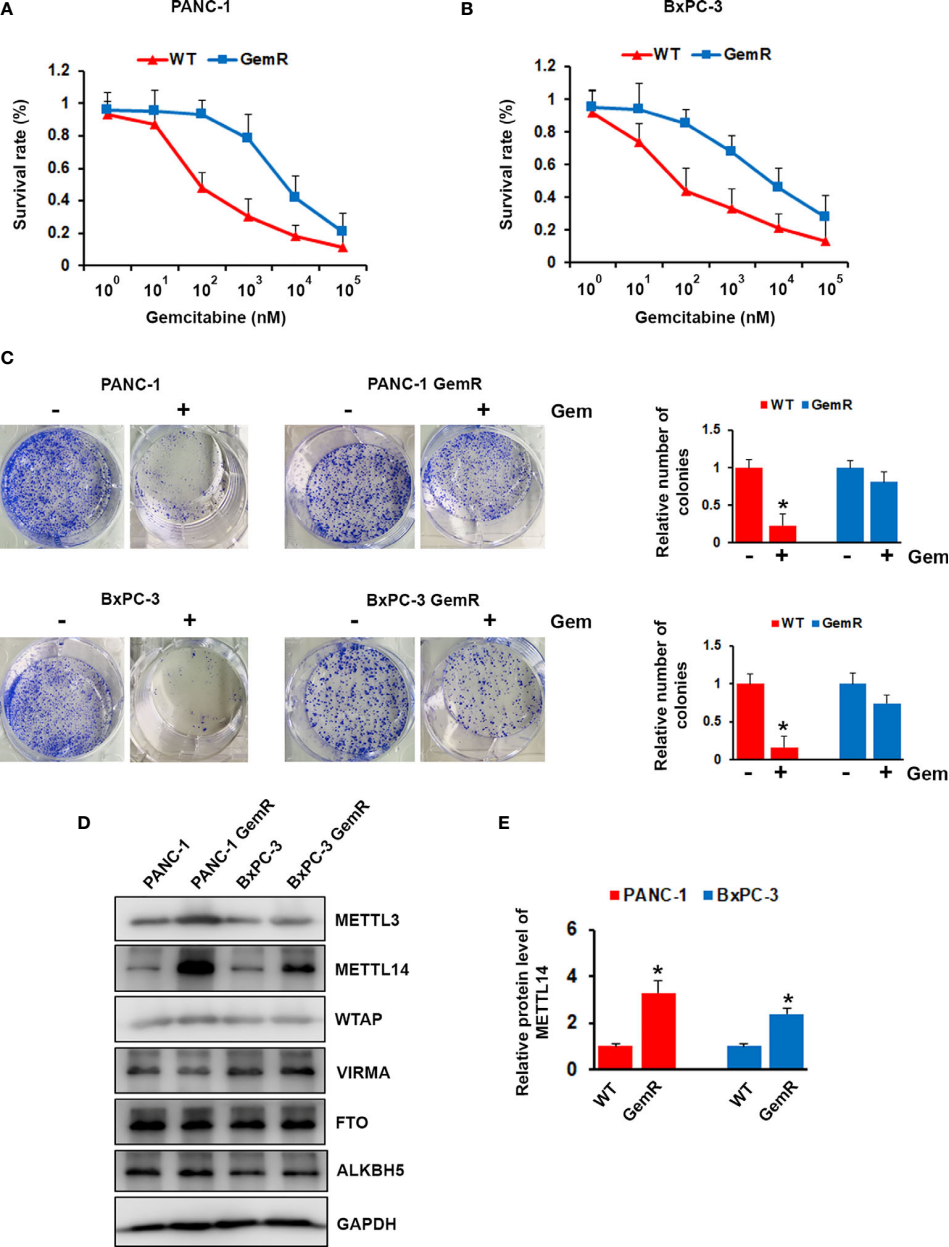
Aberrant expression of METTL14 in gemcitabine resistant pancreatic cancer cells. **(A, B)** PANC-1 GemR **(A)**/BxPC-3 GemR **(B)** and their parental wild type cells were treated with indicated concentration of gemcitabine for 72 hours, cell survival was assessed by MTT assay. **(C)** PANC-1 GemR/BxPC-3 GemR and their parental wild type cells were performed colony formation assay after treated with gemcitabine. **(D)** The protein level of METTL3, METTL14, WTAP, VIRMA, FTO, and ALKBH5 was assessed by western blotting in PANC-1 GemR/BxPC-3 GemR and their parental wild type cells. **(E)** Quantification of METTL14 protein levels in wild type (WT) or gemcitabine resistant (GemR) pancreatic cancer cells. Data are reported as means ± SEM of values from three experiments. *p < 0.05.

Resistant to the chemotherapy is the common feature of pancreatic cancer patients, which lead to poor prognosis. To investigate the expression level of m6A modification associated proteins in gemcitabine resistant pancreatic cancer cell, we generated gemcitabine resistant pancreatic cancer cell lines, PANC-1 GemR and BxPC-3 GemR. IC50 experiment showed that gemcitabine resistant (GemR) human pancreatic cancer cell lines PANC-1 and BxPC-3 showed almost 75-fold or 68-fold increase in resistance to gemcitabine compared with wild type cells (**Figures 2A, B**). Furthermore, gemcitabine treatment significantly showed inhibition on the colony formation ability of wild type PANC-1 and BxPC-3 cells, but less effect on gemcitabine resistant cells (**Figure 2C**). We next performed western blotting analysis of gemcitabine resistant PANC-1 and BxPC-3 cell lines as well as wild type cells, and indicated that the expression of METTL14 was significantly up-regulated in gemcitabine resistant cells compared with parental wild type cell lines respectively (**Figures 2D, E**).

### Expression of METTL14 Was Functionally Linked to Gemcitabine Resistance in Pancreatic Cancer Cells

**Figure 3:**
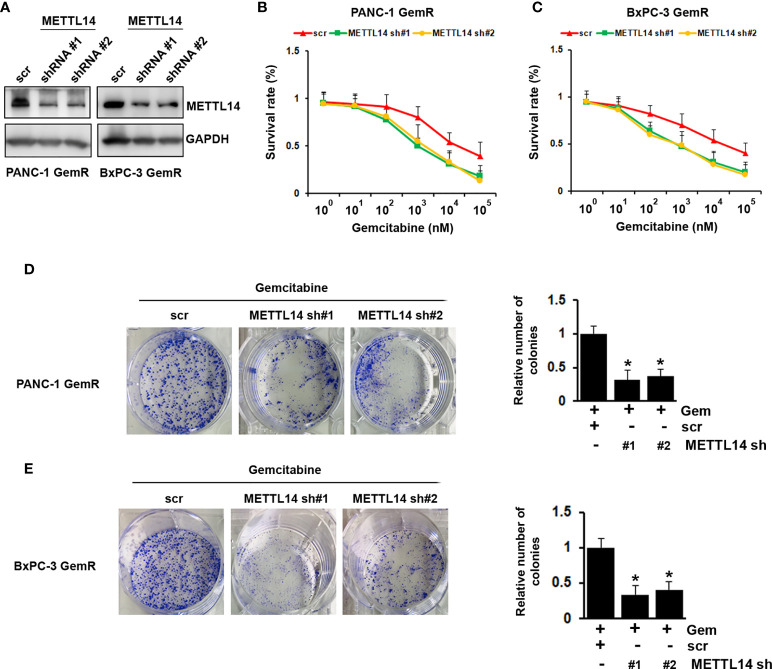
Effect of METTL14 depletion on gemcitabine resistance in pancreatic cancer cells. **(A)** The protein level of METTL14 was assessed by western blotting in PANC-1 GemR/BxPC-3 GemR cells after infected with METTL14 shRNA. **(B, C)** Gemcitabine IC50 experiment was performed in PANC-1 GemR/BxPC-3 GemR cells after infected with METTL14 shRNA. **(D, E)** PANC-1 GemR **(D)** and BxPC-3 GemR **(E)** cells were performed colony formation assay after infected with METTL14 shRNA followed with gemcitabine treatment. *p value < 0.05.

Since METTL14 was found up-regulated in gemcitabine resistant pancreatic cancer cells, we hypothesis that METTL14 has functional role in chemoresistance. To investigate that, we knock-down METTL14 in gemcitabine resistant PANC-1 and BxPC-3 cells (**Figure 3A**). Our results indicated that METTL14 depletion rescued the response of resistant cells to gemcitabine as compared to scramble transfected cells (**Figures 3B, C**). Consistently, METTL14 depletion increased the inhibitory effect of gemcitabine on colony formation ability of resistant cells (**Figures 3D, E**). Overall, our results indicated that METTL14 was up-regulated in gemcitabine resistant pancreatic cancer cell lines, and its expression was functionally linked to gemcitabine resistance.

### P65 Regulated the Expression of METTL14 in Gemcitabine Resistant Pancreatic Cancer Cells

**Figure 4:**
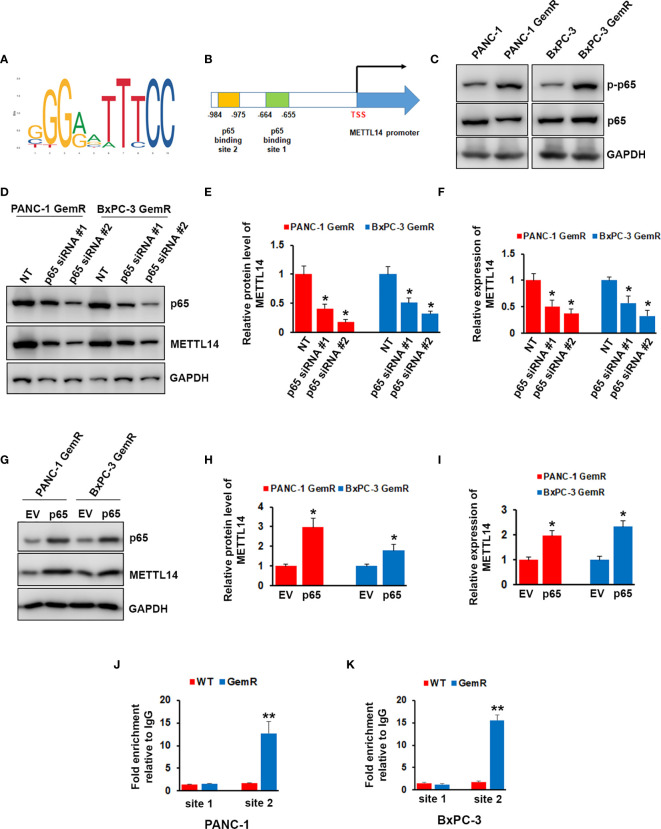
Effect of p65 on regulating METTL14 expression. **(A)** The canonical binding motif of p65. **(B)** Schematic diagram of METTL14 promoter region containing two putative p65 binding sites. **(C)** The phosphorylation of p65 and total p65 levels in PANC-1 GemR/BxPC-3 GemR and their parental wild type cells was assessed by western blotting. **(D–F)** The protein **(D, E)** and mRNA **(F)** level of METTL14 was assessed by western blotting and QRT-PCR after p65 depletion. Data are reported as means ± SEM of values from three experiments. *p < 0.05. **(G–I)** The protein **(G, H)** and mRNA **(I)** level of METTL14 was assessed by western blotting and QRT-PCR after p65 overexpression. Data are reported as means ± SEM of values from three experiments. *p < 0.05. **(J, K)** ChIP assay indicated an increase in p65 binding to METTL14 promoter *via* binding site 2 in PANC-1 GemR/BxPC-3 GemR compared with their parental wild type cells. **p < 0.01.

We next wonder how METTL14 was regulated in gemcitabine resistant pancreatic cancer cells. Transcriptional factor p65 has been shown to play important role in regulating pancreatic cancer chemoresistance (34). By analyzing METTL14 promoter sequence, we found two putative binding motif of p65 (**Figures 4A, B**). We therefore examined the total and phosphorylation levels of p65 in gemcitabine resistant cells, and our results showed that the phosphorylation level of p65 was up-regulated in both gemcitabine resistant cells, but not total level as compared to the wild type cells (**Figure 4C**). Importantly, depletion of p65 by specific shRNA significantly decreased the expression of METTL14 at mRNA and protein levels in both gemcitabine resistant cells (**Figures 4D–F**). On the contrary, p65 overexpression increased the expression of METTL14 (**Figures 4G–I**). To further confirm that p65 regulates METTL14 expression by binding its promoter, we performed chromatin immunoprecipitation (ChIP) assay using p65 antibody. As shown in **Figures 4J, K**, more p65 binding to the binding site 2 (−984~-975) was observed in both gemcitabine resistant cells compared to wild type cells. These data indicated that transcriptional factor p65 regulates the expression of METTL14 *via* binding to its promoter region.

### METTL14 Regulated the Expression of Cytidine Deaminase

**Figure 5:**
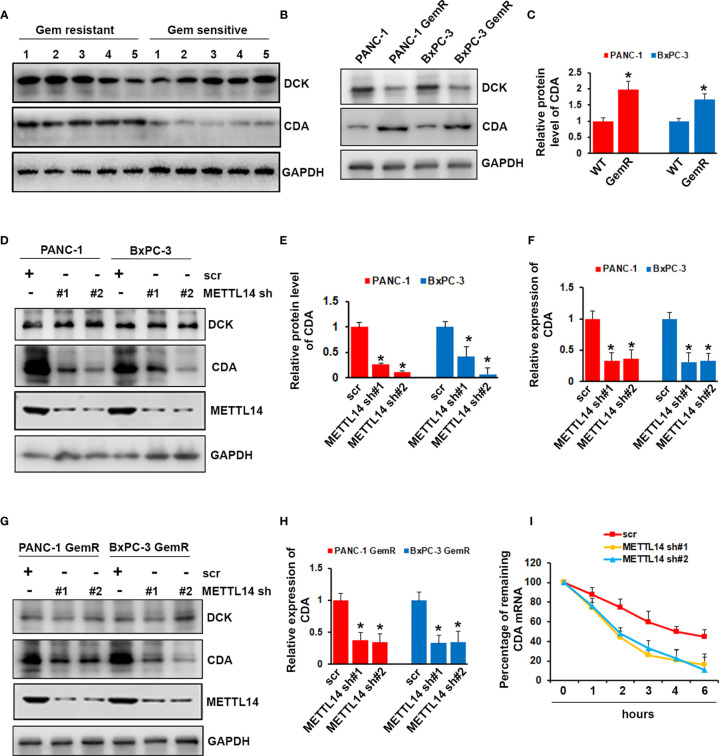
Effect of METTL14 on regulating cytidine deaminase (CDA) expression. **(A)** The protein level of DCK and CDA in gemcitabine resistant or sensitive tumors (n=5) was assessed by western blotting. **(B, C)** The protein level of DCK and CDA in PANC-1 GemR/BxPC-3 GemR and their parental wild type cells was assessed by western blotting. Data are reported as means ± SEM of values from three experiments. *p < 0.05. **(D–F)** The protein **(D, E)** and mRNA **(F)** level of CDA was assessed by western blotting and QRT-PCR after METTL14 depletion in PANC-1 and BxPC-3 cells. Data are reported as means ± SEM of values from three experiments. *p < 0.05. **(G, H)** The protein **(G)** and mRNA **(H)** level of CDA was assessed by western blotting and QRT-PCR after METTL14 depletion in PANC-1 GemR and BxPC-3 GemR cells. Data are reported as means ± SEM of values from three experiments. *p < 0.05. **(I)** QRT-PCR analysis of CDA mRNA levels in PANC-1 cells (scr or METTL14 shRNA) after actinomycin D treatment.

Although we proved that METTL14 played important role in gemcitabine resistance, the mechanism remains unclear. After uptake by cells, gemcitabine is activated by deoxycytidine kinase (DCK) and inactivated by cytidine deaminase (CDA). We wonder whether METTL14 could regulate the expression level of DCK or CDA. First, we compared the expression level of DCK and CDA in gemcitabine resistant and sensitive tumors (n=5), and we found that CDA was highly expressed in gemcitabine resistant tumors while no significant difference of DCK expression was observed (**Figure 5A**). We next examined the expression level of DCK and CDA in gemcitabine resistant and wild type cells. As shown in **Figures 5B, C**, DCK was decreased, while CDA was increased in gemcitabine resistant cells compared with wild type cells. However, METTL14 depletion only decreased the mRNA and protein level of CDA, but had no effect on DCK in pancreatic cancer cells (**Figures 5D–F**). Similar result was also observed in gemcitabine resistant pancreatic cancer cells (**Figures 5G, H**). More importantly, METTL14 depletion led to a marked decrease in the CDA transcript half-life after treatment with the transcriptional inhibitor actinomycin D, which indicated that METTL14 regulated the stability of CDA transcripts (**Figure 5I**). These results suggested that METTL14 promotes gemcitabine resistance *via* regulating CDA expression.

### METTL14 Deletion Increased Gemcitabine Sensitivity *In Vivo*

**Figure 6:**
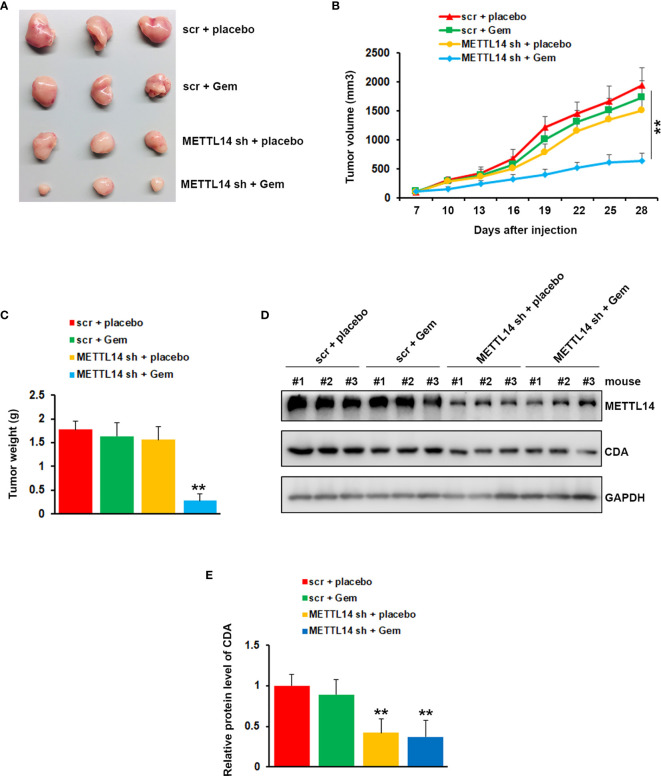
Effects of METTL14 depletion on gemcitabine resistance *in vivo*. **(A)** Typical photos of tumors from each group. **(B, C)** Tumor growth curve **(B)** and weight **(C)** indicated that METTL14 depletion significantly rescued the response of PANC-1 GemR cells to gemcitabine. **p < 0.01. **(D)** Protein level of CDA and METTL14 in 3 representative tumors from each group were analyzed by western blotting. **(E)** Quantification of CDA protein levels in 3 representative tumors from each group. **p < 0.01.

To further confirm the critical role of METTL14 in gemcitabine resistance, we tested the function of METTL14 depletion on drug sensitivity in a mouse model. The mouse model was established by injecting PANC-1 GemR cells infected with Scr or METTL14 shRNA into NOD/SCID mice. One week after injection, the mice were treated with vehicle or 100 mg/kg gemcitabine intraperitoneally twice a week. In agreement with our *in vitro* data, the mice bearing PANC-1 GemR cells infected with scramble shRNA did not response to gemcitabine treatment, whereas depletion of METTL14 showed greater response to gemcitabine as compared to the vehicle treated group (**Figures 6A, B**). Tumor weights were also significantly lower in gemcitabine treated group as compared to the tumors harvested from control group after METTL14 depletion (**Figure 6C**). To examine the impact of METTL14 depletion on CDA, 3 representative tumors from each group were analyzed by using western blotting. As shown in **Figures 6D, E**, the CDA protein levels in tumors harvested from animals bearing PANC-1 GemR cells infected with METTL14 shRNA were significantly lower as compared to tissues from scramble groups. Taken together these results indicated that METTL14 depletion increased the sensitivity of resistant cells to gemcitabine treatment.

## Discussion

Pancreatic cancer is a devastating disease and only 15-20% patients are suitable for surgery (35). Intrinsic and acquired chemotherapy resistance, especially gemcitabine, is the major obstacle for the successful therapy of pancreatic cancer. Recently, an increasing number of studies have been indicating that abnormal epigenetic regulation of gene function plays an important role in drug resistance. Many research showed that epigenetic drugs circumvent resistance to conventional therapeutics in different tumor (36). Among epigenetic regulation, m6A methylation has been demonstrated as the most common type of RNA modification. The precisely regulated m6A modifications play an extremely important role in maintenance of biological activities. m6A deregulation happened in cancer affect tumor oncogene and suppressor signal pathway *via* alteration of RNA translation efficiency and stability depending on the cellular environment (37). As one of the key methyltransferase, METTL14 is a scaffold for RNA binding, identifies the substrate of the m6A methyltransferase complex, and shares about 20% sequence identity with domains found in METTL3. In the m6A methyltransferase complex, METTL14 is thought to assume a pseudomethyltransferase function that helps bind RNA and stabilize METTL3 (38).

Recently, the function of m6A modification in various cancers, including bladder, breast, liver, lung, leukemia, brain, cervical, and endometrial cancer, has been revealed (19, 39). In pancreatic cancer, the m6A eraser ALKBH5 was not only considered as a tumor-suppressor gene, which was involved in sensitizing pancreatic cancer cells to chemotherapy *via* regulating Wnt signaling, but also reported to prevent pancreatic cancer progression by posttranscriptional activation of PER1 in an YTHDF2-dependent manner (27, 40). The genetic variations in the FTO gene are associated with pancreatic cancer risk through a possible mechanism that is independent of obesity (24). METTL3 was reported to promote cancer progression and increase chemo, radio, and chemoradio-sensitivity in pancreatic cancer (31). In addition, Wang et al. demonstrated the critical role of METTL14 in the growth and metastasis of pancreatic cancer *via* targeting of PERP mRNA (28). In the present study, we demonstrate that the expression of METTL14 was increased after gemcitabine treatment, and METTL14, but not other m6A associated proteins, was overexpressed in the gemcitabine resistant pancreatic cancer cell lines. Depletion of METTL14 significantly increased the sensitivity of resistant pancreatic cells to gemcitabine, which suggested the critical role of METTL14 in gemcitabine resistance.

Although the expression of METTL14 was increased in the resistant pancreatic cancer cells, the regulatory mechanism of expression remains unclear. Transcriptional factor p65 has been reported to link to gemcitabine resistance, and elevated p65 activity was found in gemcitabine resistant pancreatic cancer cell line-MIA Paca2 (41, 42). Thus, we wonder whether p65 could regulate the expression of METTL14. Interestingly, we found two putative p65 binding motif on METTL14 promoter region after analyzing the promoter sequence of METTL14. Consistent with other’s report (42), we found phosphorylation level of p65 was increased in our gemcitabine resistant cell lines. Inhibition of METTL14 by siRNA knockdown significantly decreased the expression level of METTL14, while overexpressing p65 increased METTL14 expression. More importantly, our ChIP assay showed elevated p65 binding on METTL14 promoter region. Altogether, our results suggested that p65 is the regulator of METTL14 expression in gemcitabine resistant pancreatic cells.

Gemcitabine, a deoxycytidine nucleoside analog, is activated by phosphorylation and then inhibits DNA synthesis, blocks cell cycle, and attenuates cell proliferation. After uptake by cells, gemcitabine is activated by DCK and inactivated by CDA. In tumor cells, DCK inhibition causes gemcitabine resistance, while CDA suppression results in gemcitabine chemosensitivity. We found that DCK was decreased and CDA was overexpressed in gemcitabine resistant tumor samples and pancreatic cancer cells. However, DCK was only found deregulated in gemcitabine resistant pancreatic cancer cells, but not tumor samples. Interestingly, METTL14 knockdown decreased CDA level, but had no effect on DCK level. m^6^A marks on mRNA transcripts was reported to affect mRNA stability (43). To investigate whether METTL14 regulates CDA* *expression *via* regulating its mRNA stability, we treated control or METTL14 knockdown PANC-1 cells with the transcription inhibitor actinomycin D (Act D) and detected the half-lives of CDA transcripts. As shown in **Figure 5I**, METTL14 depletion resulted in a noticeable decrease in the half-lives of CDA transcripts. These results indicated that CDA is the downstream target of METTL14 in gemcitabine resistant pancreatic cancer cells.

## Conclusion

We have determined the role of METTL14 in promoting the resistance of gemcitabine in pancreatic cancer cells. Mechanistically, p65 binds the promoter region of METTL14 and increases the expression of METTL14, thus elevates the CDA level, which consequently inactivates gemcitabine. The results of this study provide potential therapeutic target and method for pancreatic cancer patients with gemcitabine resistance.

## Data Availability Statement

The original contributions presented in the study are included in the article/supplementary material. Further inquiries can be directed to the corresponding authors.

## Ethics Statement

The studies involving human participants were reviewed and approved by The Ethics Committee of the First Affiliated Hospital of Anhui Medical University. Written informed consent for participation was not required for this study in accordance with the national legislation and the institutional requirements. The animal study was reviewed and approved by the Ethics Committee of the First Affiliated Hospital of Anhui Medical University.

## Author Contributions

YL and RX contributed to the conception and design of the study. CZ, SO, and YZ performed the statistical analysis, the experimental operation and organized the experimental data. YZ, CZ, and SO wrote the first draft of the manuscript. PL, PZ, and ZL contributed to data collection and some of the experiments. All authors contributed to the article and approved the submitted version.

## Conflict of Interest

The authors declare that the research was conducted in the absence of any commercial or financial relationships that could be construed as a potential conflict of interest.

## Publisher’s Note

All claims expressed in this article are solely those of the authors and do not necessarily represent those of their affiliated organizations, or those of the publisher, the editors and the reviewers. Any product that may be evaluated in this article, or claim that may be made by its manufacturer, is not guaranteed or endorsed by the publisher.

## References

[1] Middleton G Palmer DH Greenhalf W Ghaneh P Jackson R Cox T. Vandetanib Plus Gemcitabine Versus Placebo Plus Gemcitabine in Locally Advanced or Metastatic Pancreatic Carcinoma (ViP): A Prospective, Randomised, Double-Blind, Multicentre Phase 2 Trial. Lancet Oncol (2017) 18(4):486–99. 10.1016/S1470-2045(17)30084-0 28259610

[2] Sung H Ferlay J Siegel RL Laversanne M Soerjomataram I Jemal A. Global Cancer Statistics 2020: GLOBOCAN Estimates of Incidence and Mortality Worldwide for 36 Cancers in 185 Countries. CA Cancer J Clin (2021) 71(3):209–49. 10.3322/caac.21660 33538338

[3] Kamisawa T Wood LD Itoi T Takaori K. Pancreatic Cancer. Lancet (2016) 388(10039):73–85. 10.1016/S0140-6736(16)00141-0 26830752

[4] Neoptolemos JP Stocken DD Bassi C Ghaneh P Cunningham D Goldstein D. Adjuvant Chemotherapy With Fluorouracil Plus Folinic Acid vs Gemcitabine Following Pancreatic Cancer Resection: A Randomized Controlled Trial. JAMA (2010) 304(10):1073–81. 10.1001/jama.2010.1275 20823433

[5] Oettle H Neuhaus P Hochhaus A Hartmann JT Gellert K Ridwelski K. Adjuvant Chemotherapy With Gemcitabine and Long-Term Outcomes Among Patients With Resected Pancreatic Cancer: The CONKO-001 Randomized Trial. JAMA (2013) 310(14):1473–81. 10.1001/jama.2013.279201 24104372

[6] Conroy T Desseigne F Ychou M Bouche O Guimbaud R Becouarn Y. FOLFIRINOX Versus Gemcitabine for Metastatic Pancreatic Cancer. N Engl J Med (2011) 364(19):1817–25. 10.1056/NEJMoa1011923 21561347

[7] Sohal DP Mangu PB Khorana AA Shah MA Philip PA O’Reilly EM. Metastatic Pancreatic Cancer: American Society of Clinical Oncology Clinical Practice Guideline. J Clin Oncol Off J Am Soc Clin Oncol (2016) 34(23):2784–96. 10.1200/JCO.2016.67.1412 PMC5019760 27247222

[8] Oettle H Post S Neuhaus P Gellert K Langrehr J Ridwelski K. Adjuvant Chemotherapy With Gemcitabine vs Observation in Patients Undergoing Curative-Intent Resection of Pancreatic Cancer: A Randomized Controlled Trial. JAMA (2007) 297(3):267–77. 10.1001/jama.297.3.267 17227978

[9] Sarvepalli D Rashid MU Rahman AU Ullah W Hussain I Hasan B. Gemcitabine: A Review of Chemoresistance in Pancreatic Cancer. Crit Rev Oncog (2019) 24(2):199–212. 10.1615/CritRevOncog.2019031641 31679214

[10] Bjanes TK Jordheim LP Schjott J Kamceva T Cros-Perrial E Langer A. Intracellular Cytidine Deaminase Regulates Gemcitabine Metabolism in Pancreatic Cancer Cell Lines. Drug Metab Dispos (2020) 48(3):153–8. 10.1124/dmd.119.089334 PMC11022907 31871136

[11] Oettle H Lehmann T. Gemcitabine-Resistant Pancreatic Cancer: A Second-Line Option. Lancet (2016) 387(10018):507–8. 10.1016/S0140-6736(15)01035-1 26616909

[12] Desrosiers R Friderici K Rottman F. Identification of Methylated Nucleosides in Messenger RNA From Novikoff Hepatoma Cells. Proc Natl Acad Sci USA (1974) 71(10):3971–5. 10.1073/pnas.71.10.3971 PMC434308 4372599

[13] Wei CM Gershowitz A Moss B. Methylated Nucleotides Block 5’ Terminus of HeLa Cell Messenger RNA. Cell (1975) 4(4):379–86. 10.1016/0092-8674(75)90158-0 164293

[14] Min KW Zealy RW Davila S Fomin M Cummings JC Makowsky D. Profiling of M6a RNA Modifications Identified an Age-Associated Regulation of AGO2 mRNA Stability. Aging Cell (2018) 17(3):e12753. 10.1111/acel.12753 29573145 PMC5946072

[15] Ma S Chen C Ji X Liu J Zhou Q Wang G. The Interplay Between M6a RNA Methylation and Noncoding RNA in Cancer. J Hematol Oncol (2019) 12(1):121. 10.1186/s13045-019-0805-7 31757221 PMC6874823

[16] Ping XL Sun BF Wang L Xiao W Yang X Wang WJ. Mammalian WTAP Is a Regulatory Subunit of the RNA N6-Methyladenosine Methyltransferase. Cell Res (2014) 24(2):177–89. 10.1038/cr.2014.3 PMC3915904 24407421

[17] Jia G Fu Y Zhao X Dai Q Zheng G Yang Y. N6-Methyladenosine in Nuclear RNA Is a Major Substrate of the Obesity-Associated FTO. Nat Chem Biol (2011) 7(12):885–7. 10.1038/nchembio.687 PMC3218240 22002720

[18] Zheng G Dahl JA Niu Y Fedorcsak P Huang CM Li CJ. ALKBH5 Is a Mammalian RNA Demethylase That Impacts RNA Metabolism and Mouse Fertility. Mol Cell (2013) 49(1):18–29. 10.1016/j.molcel.2012.10.015 23177736 PMC3646334

[19] Chen XY Zhang J Zhu JS. The Role of M(6)a RNA Methylation in Human Cancer. Mol Cancer (2019) 18(1):103. 10.1186/s12943-019-1033-z 31142332 PMC6540575

[20] Dominissini D Moshitch-Moshkovitz S Schwartz S Salmon-Divon M Ungar L Osenberg S. Topology of the Human and Mouse M6a RNA Methylomes Revealed by M6a-Seq. Nature (2012) 485(7397):201–6. 10.1038/nature11112 22575960

[21] Li Z Weng H Su R Weng X Zuo Z Li C. Fto Plays an Oncogenic Role in Acute Myeloid Leukemia as a N(6)-Methyladenosine RNA Demethylase. Cancer Cell (2017) 31(1):127–41. 10.1016/j.ccell.2016.11.017 PMC5234852 28017614

[22] Audzevich T Bashford-Rogers R Mabbott NA Frampton D Freeman TC Potocnik A. Pre/Pro-B Cells Generate Macrophage Populations During Homeostasis and Inflammation. Proc Natl Acad Sci USA (2017) 114(20):E3954–63. 10.1073/pnas.1616417114 PMC5441795 28461481

[23] Chen M Wei L Law CT Tsang FH Shen J Cheng CL. RNA N6-methyladenosine Methyltransferase-Like 3 Promotes Liver Cancer Progression Through YTHDF2-dependent Posttranscriptional Silencing of SOCS2. Hepatology (2018) 67(6):2254–70. 10.1002/hep.29683 29171881

[24] Lin Y Ueda J Yagyu K Ishii H Ueno M Egawa N. Association Between Variations in the Fat Mass and Obesity-Associated Gene and Pancreatic Cancer Risk: A Case-Control Study in Japan. BMC Cancer (2013) 13:337. 10.1186/1471-2407-13-337 23835106 PMC3716552

[25] Chen J Sun Y Xu X Wang D He J Zhou H. YTH Domain Family 2 Orchestrates Epithelial-Mesenchymal Transition/Proliferation Dichotomy in Pancreatic Cancer Cells. Cell Cycle (2017) 16(23):2259–71. 10.1080/15384101.2017.1380125 PMC5788481 29135329

[26] Xia T Wu X Cao M Zhang P Shi G Zhang J. The RNA m6A Methyltransferase METTL3 Promotes Pancreatic Cancer Cell Proliferation and Invasion. Pathol Res Pract (2019) 215(11):152666. 10.1016/j.prp.2019.152666 31606241

[27] Guo X Li K Jiang W Hu Y Xiao W Huang Y. RNA Demethylase ALKBH5 Prevents Pancreatic Cancer Progression by Posttranscriptional Activation of PER1 in an M6a-YTHDF2-dependent Manner. Mol Cancer (2020) 19(1):91. 10.1186/s12943-020-01158-w 32429928 PMC7236181

[28] Wang M Liu J Zhao Y He R Xu X Guo X. Upregulation of METTL14 Mediates the Elevation of PERP mRNA N(6) Adenosine Methylation Promoting the Growth and Metastasis of Pancreatic Cancer. Mol Cancer (2020) 19(1):130. 10.1186/s12943-020-01249-8 32843065 PMC7446161

[29] Yan F Al-Kali A Zhang Z Liu J Pang J Zhao N. A Dynamic N(6)-methyladenosine Methylome Regulates Intrinsic and Acquired Resistance to Tyrosine Kinase Inhibitors. Cell Res (2018) 28(11):1062–76. 10.1038/s41422-018-0097-4 PMC6218444 30297871

[30] Xiang M Liu W Tian W You A Deng D. RNA N-6-methyladenosine Enzymes and Resistance of Cancer Cells to Chemotherapy and Radiotherapy. Epigenomics (2020) 12(9):801–9. 10.2217/epi-2019-0358 32515221

[31] Taketo K Konno M Asai A Koseki J Toratani M Satoh T. The Epitranscriptome m6A Writer METTL3 Promotes Chemo- and Radioresistance in Pancreatic Cancer Cells. Int J Oncol (2018) 52(2):621–9. 10.3892/ijo.2017.4219 29345285

[32] Ballard DW Dixon EP Peffer NJ Bogerd H Doerre S Stein B. The 65-kDa Subunit of Human NF-Kappa B Functions as a Potent Transcriptional Activator and a Target for V-Rel-Mediated Repression. Proc Natl Acad Sci USA (1992) 89(5):1875–9. 10.1073/pnas.89.5.1875 PMC48556 1542686

[33] Weidensdorfer D Stohr N Baude A Lederer M Kohn M Schierhorn A. Control of C-Myc mRNA Stability by IGF2BP1-Associated Cytoplasmic Rnps. RNA (2009) 15(1):104–15. 10.1261/rna.1175909 PMC2612774 19029303

[34] Meng Q Liang C Hua J Zhang B Liu J Zhang Y. A miR-146a-5p/TRAF6/NF-kB p65 Axis Regulates Pancreatic Cancer Chemoresistance: Functional Validation and Clinical Significance. Theranostics (2020) 10(9):3967–79. 10.7150/thno.40566 PMC7086345 32226532

[35] Shaib WL Ip A Cardona K Alese OB Maithel SK Kooby D. Contemporary Management of Borderline Resectable and Locally Advanced Unresectable Pancreatic Cancer. Oncologist (2016) 21(2):178–87. 10.1634/theoncologist.2015-0316 PMC4746088 26834159

[36] Rauscher S Greil R Geisberger R. Re-Sensitizing Tumor Cells to Cancer Drugs With Epigenetic Regulators. Curr Cancer Drug Targets (2021) 21(4):353–9. 10.2174/1568009620666210108102723 33423645

[37] Wang X Zhao BS Roundtree IA Lu Z Han D Ma H. N(6)-Methyladenosine Modulates Messenger RNA Translation Efficiency. Cell (2015) 161(6):1388–99. 10.1016/j.cell.2015.05.014 PMC4825696 26046440

[38] Wang X Huang J Zou T Yin P. Human M(6)a Writers: Two Subunits, 2 Roles. RNA Biol (2017) 14(3):300–4. 10.1080/15476286.2017.1282025 PMC5367249 28121234

[39] Choe J Lin S Zhang W Liu Q Wang L Ramirez-Moya J. mRNA Circularization by METTL3-eIF3h Enhances Translation and Promotes Oncogenesis. Nature (2018) 561(7724):556–60. 10.1038/s41586-018-0538-8 PMC6234840 30232453

[40] Tang B Yang Y Kang M Wang Y Wang Y Bi Y. M(6)a Demethylase ALKBH5 Inhibits Pancreatic Cancer Tumorigenesis by Decreasing WIF-1 RNA Methylation and Mediating Wnt Signaling. Mol Cancer (2020) 19(1):3. 10.1186/s12943-019-1128-6 31906946 PMC6943907

[41] Pan X Arumugam T Yamamoto T Levin PA Ramachandran V Ji B. Nuclear Factor-Kappab p65/relA Silencing Induces Apoptosis and Increases Gemcitabine Effectiveness in a Subset of Pancreatic Cancer Cells. Clin Cancer Res (2008) 14(24):8143–51. 10.1158/1078-0432.CCR-08-1539 PMC4403242 19088029

[42] Chen Y Su L Huang C Wu S Qiu X Zhao X. Galactosyltransferase B4GALT1 Confers Chemoresistance in Pancreatic Ductal Adenocarcinomas by Upregulating N-linked Glycosylation of CDK11(P110). Cancer Lett (2021) 500:228–43. 10.1016/j.canlet.2020.12.006 33309857

[43] Weng H Huang H Wu H Qin X Zhao BS Dong L. Mettl14 Inhibits Hematopoietic Stem/Progenitor Differentiation and Promotes Leukemogenesis *Via* mRNA M(6)a Modification. Cell Stem Cell (2018) 22(2):191–205.e9. 10.1016/j.stem.2017.11.016 29290617 PMC5860916

